# Supplementary material for: Empirical methods for controlling false positives and estimating confidence in ChIP-Seq peaks
Source: BMC Bioinformatics. 2008 Dec 5;9:523. doi: 10.1186/1471-2105-9-523 (PMC2628906; doi:10.1186/1471-2105-9-523)
Supplement: Additional File 1 — A variety of html documents from the USeq web site detailing the available applications, their best usage, output file type descriptions, command line menus, etc. [file 1471-2105-9-523-S1.zip › USeqUserGuides/JavaDoc.zip!/JavaDoc/util/bio/calc/NucleicAcid.html]

NucleicAcid


|  |  |  |
| --- | --- | --- |
| **Overview** \| **Package** \| **Class** \| **Use** \| **Tree** \| **Deprecated** \| **Index** \| **Help** | |  |
| **PREV CLASS**   NEXT CLASS | **FRAMES**    **NO FRAMES**     **All Classes** |  |
| SUMMARY: NESTED \| FIELD \| CONSTR \| METHOD | DETAIL: FIELD \| CONSTR \| METHOD |  |


---


## util.bio.calc Class NucleicAcid

```
java.lang.Object
  util.bio.calc.NucleicAcid
```

---

``` public class NucleicAcid extends java.lang.Object ```

Nucleic Acid parameter calculator and converter static methods.

---

| **Field Summary** | |
| --- | --- |
| `static double[][]` | `dH`             delta H values for dinucleotide pairs. |
| `static double[][]` | `dS`             delta S values for dinucleotide pairs. |


**Constructor Summary**

`NucleicAcid()`


| **Method Summary** | |
| --- | --- |
| `static int` | `baseNumber(char b)`             Returns 0 for a, 1 for c, 2 for g, 3 for t, -1 for everything else. |
| `static double` | `calcDefaultNearestNeighborTm(java.lang.String seq)`             Calculates a nearest neighbor Oligo Tm at 50nM oligo, 50mM Salt, also corrects case for real method. |
| `static double` | `calculateBasicOligoTm(java.lang.String seq)`             Calculate basic Oligo Tm |
| `static double` | `calculateFractionGC(java.lang.String DNA)`             Calculates a GC concentration, case insensitive. |
| `static double` | `calculateMolecularWtDNA(int[] gatcn)`             Calculates molecular weight of a DNA sequence given an int[] of gatcn's, no 5' phosphate |
| `static double` | `calculateMolecularWtRNA(int[] gaucn)`             Calculates molecular weight of a RNA sequence given an int[] of gaucn's, no 5' phosphate |
| `static double` | `calculateSaltAdjustedOligoTm(java.lang.String seq)`             Calculate salt adjusted Oligo Tm |
| `static double` | `convertToMicroMolarDNA(java.lang.String seq, double ngPerUl)`             coverts ng/ul to uM given a DNA sequence |
| `static double` | `convertToMicroMolarRNA(java.lang.String seq, double ngPerUl)`             coverts ng/ul to uM given an RNA sequence |
| `static double` | `convertToNgPerUlDNA(java.lang.String seq, double uM)`             coverts uM to ng/ul given a DNA sequence |
| `static double` | `convertToNgPerUlRNA(java.lang.String seq, double uM)`             coverts uM to ng/ul given an RNA sequence |
| `static java.lang.String` | `formatNumberOneFraction(double num)`             Takes a double returns a String with one fractional digit 9.54443 -> 9.5 |
| `static java.lang.String` | `formatPercentOneFraction(double num)`             Takes a double in percent returns a String with one fraction 0.85842 -> 85.8% |
| `static double` | `getTotalDelta(double[][] delta, java.lang.String seq)`             Returns the total delta given a lower case DNA sequence. |
| `static double` | `nearestNeighborOligoTm(java.lang.String seqLowerCase, double oligoConcentration, double saltConcentration)`             Calculates nearest neighbor tm given a DNA oligonucleotide. |

**Methods inherited from class java.lang.Object**

`equals, getClass, hashCode, notify, notifyAll, toString, wait, wait, wait`

**Field Detail**

### dH

```
public static final double[][] dH
```

:   delta H values for dinucleotide pairs.
    For tmNN().

---


### dS

```
public static final double[][] dS
```

:   delta S values for dinucleotide pairs.
    For tmNN().


**Constructor Detail**

### NucleicAcid

```
public NucleicAcid()
```


**Method Detail**

### convertToMicroMolarDNA

```
public static double convertToMicroMolarDNA(java.lang.String seq,
                                            double ngPerUl)
```

:   coverts ng/ul to uM given a DNA sequence

---


### convertToNgPerUlDNA

```
public static double convertToNgPerUlDNA(java.lang.String seq,
                                         double uM)
```

:   coverts uM to ng/ul given a DNA sequence

---


### convertToMicroMolarRNA

```
public static double convertToMicroMolarRNA(java.lang.String seq,
                                            double ngPerUl)
```

:   coverts ng/ul to uM given an RNA sequence

---


### convertToNgPerUlRNA

```
public static double convertToNgPerUlRNA(java.lang.String seq,
                                         double uM)
```

:   coverts uM to ng/ul given an RNA sequence

---


### calculateMolecularWtDNA

```
public static double calculateMolecularWtDNA(int[] gatcn)
```

:   Calculates molecular weight of a DNA sequence given an int[] of gatcn's, no 5' phosphate

---


### calculateMolecularWtRNA

```
public static double calculateMolecularWtRNA(int[] gaucn)
```

:   Calculates molecular weight of a RNA sequence given an int[] of gaucn's, no 5' phosphate

---


### calculateFractionGC

```
public static double calculateFractionGC(java.lang.String DNA)
```

:   Calculates a GC concentration, case insensitive.

---


### formatPercentOneFraction

```
public static java.lang.String formatPercentOneFraction(double num)
```

:   Takes a double in percent returns a String with one fraction 0.85842 -> 85.8%

---


### formatNumberOneFraction

```
public static java.lang.String formatNumberOneFraction(double num)
```

:   Takes a double returns a String with one fractional digit 9.54443 -> 9.5

---


### calculateBasicOligoTm

```
public static double calculateBasicOligoTm(java.lang.String seq)
```

:   Calculate basic Oligo Tm

---


### calcDefaultNearestNeighborTm

```
public static double calcDefaultNearestNeighborTm(java.lang.String seq)
```

:   Calculates a nearest neighbor Oligo Tm at 50nM oligo, 50mM Salt, also corrects case
    for real method.

---


### calculateSaltAdjustedOligoTm

```
public static double calculateSaltAdjustedOligoTm(java.lang.String seq)
```

:   Calculate salt adjusted Oligo Tm

---


### nearestNeighborOligoTm

```
public static double nearestNeighborOligoTm(java.lang.String seqLowerCase,
                                            double oligoConcentration,
                                            double saltConcentration)
```

:   Calculates nearest neighbor tm given a DNA oligonucleotide.
    Concentrations are in moles, suggest 0.00000005 for oligo (50nM) and 0.05 salt (50mM).
    Lower case is faster, only gatcGATC, nothing else.
    Tm = {(DH-3.4)/(DS+1.9872ln(1/[primer])} + 16.6\*log10([Na+]) �273.15

---


### getTotalDelta

```
public static double getTotalDelta(double[][] delta,
                                   java.lang.String seq)
```

:   Returns the total delta given a lower case DNA sequence.
    For tmNN().

---


### baseNumber

```
public static int baseNumber(char b)
```

:   Returns 0 for a, 1 for c, 2 for g, 3 for t, -1 for everything else.
    For tmNN().


---


|  |  |  |
| --- | --- | --- |
| **Overview** \| **Package** \| **Class** \| **Use** \| **Tree** \| **Deprecated** \| **Index** \| **Help** | |  |
| **PREV CLASS**   NEXT CLASS | **FRAMES**    **NO FRAMES**     **All Classes** |  |
| SUMMARY: NESTED \| FIELD \| CONSTR \| METHOD | DETAIL: FIELD \| CONSTR \| METHOD |  |


---
